# Kruppel-like Factors in Skeletal Physiology and Pathologies

**DOI:** 10.3390/ijms232315174

**Published:** 2022-12-02

**Authors:** Makoto Abe, Naoya Saeki, Yuki Ikeda, Shinsuke Ohba

**Affiliations:** 1 Department of Oral Anatomy and Developmental Biology, Osaka University Graduate School of Dentistry, 1-8 Yamada-oka, Suita 565-0871, Osaka, Japan; 2 Division of Special Care Dentistry, Osaka University Dental Hospital, 1-8 Yamada-oka, Suita 565-0871, Osaka, Japan; 3 Department of Applied Prosthodontics, Graduate School of Biomedical Sciences, Nagasaki University, 1-7-1 Sakamoto, Nagasaki 852-8521, Nagasaki, Japan

**Keywords:** Kruppel-like factor, osteoblast, osteoclast, osteoarthritis, osteoporosis, osteosarcoma

## Abstract

Kruppel-like factors (KLFs) belong to a large group of zinc finger-containing transcription factors with amino acid sequences resembling the Drosophila gap gene *Krüppel*. Since the first report of molecular cloning of the *KLF* family gene, the number of KLFs has increased rapidly. Currently, 17 murine and human KLFs are known to play crucial roles in the regulation of transcription, cell proliferation, cellular differentiation, stem cell maintenance, and tissue and organ pathogenesis. Recent evidence has shown that many KLF family molecules affect skeletal cells and regulate their differentiation and function. This review summarizes the current understanding of the unique roles of each KLF in skeletal cells during normal development and skeletal pathologies.

## 1. Skeletal Development and Maintenance

Skeletons play a crucial role in the physical support of the body. Subsequent to gastrulation, the notochord is formed, which is a cartilaginous shaft in the growing fetus, providing physical support to the body. In the late stages of embryogenesis, numerous bones and cartilage develop to accommodate the acute expansion of body size. Several cell types, including osteoblasts, chondrocytes, osteoclasts, osteocytes, and vascular endothelial cells, cooperate to generate skeletal tissue, of which bone-forming osteoblasts and bone-resorbing osteoclasts are the two main cellular constituents [1].

Osteoprogenitor cells arise from multiple anatomical sites during embryogenesis. Osteoblasts in the craniofacial regions originate from neural crest cells derived from the dorsal neural ectoderm [2]. In contrast, axial and appendicular long bones originate from the paraxial mesoderm and lateral plate mesoderm, respectively [3]. Bones also have the unique ability to regenerate after injury and are restored to the pre-injury state in most cases. Postnatal skeletal stem cells play an important role in bone repair [4]. Skeletal stem cells can differentiate into bone, cartilage, and bone stroma, and also support hematopoiesis [5,6]. The differentiation of bone cells relies on the intrinsic activation of specific transcription factors at specific moments and sites. Crucial transcription factors, including SRY-box transcription factor 9 (SOX9), runt-related transcription factor 2 (RUNX2), osterix (SP7), nuclear factor of activated T-cells (NFATc1), nuclear factor-κB (NF-kB), and strawberry notch homolog 2 (SBNO2), have been identified in the timely differentiation of bone-forming and bone-resorbing cells [4]. Accumulating evidence has demonstrated that Kruppel-like factor (KLF), a vertebrate homologue of Drosophila Krüppel, plays vital roles in bone cell differentiation and skeletal maintenance. This review summarizes the current knowledge of KLFs in skeletal physiology and pathologies.

## 2. Krüppel in Drosophila

**Figure 1:**
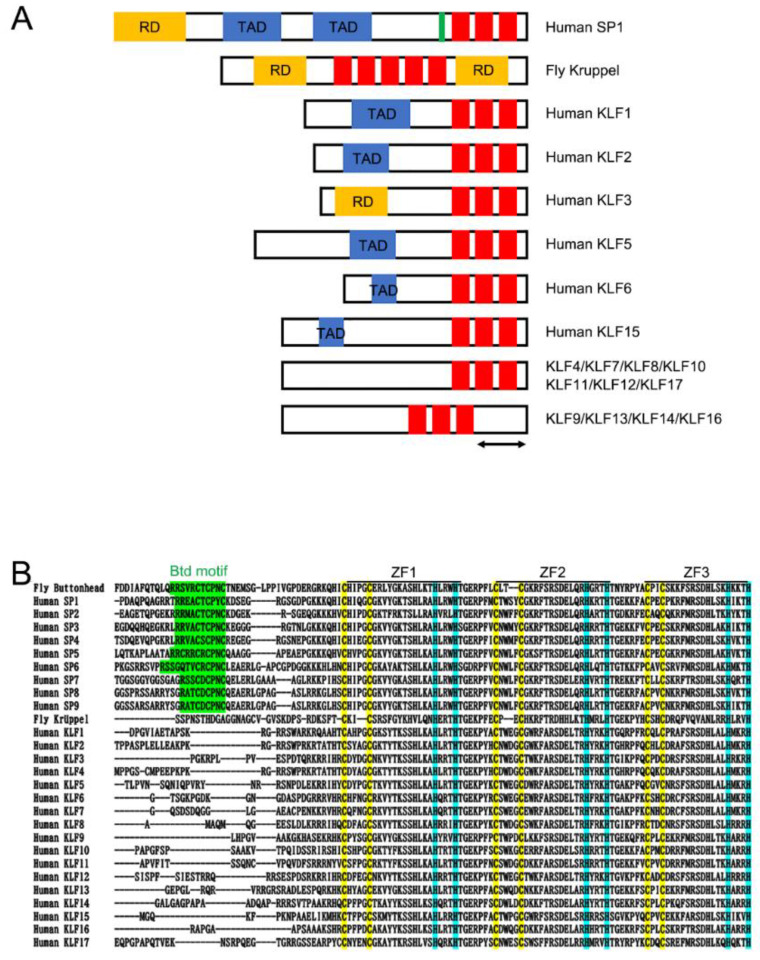
Structural and amino acid sequence properties of KLF proteins. (**A**) Structure of human SP1, Drosophila melanogaster (Fly) Krüppel, and human KLF proteins. TAD: transcriptional activation domain, RD: repression domain. The green region in SP1 protein defines the Buttonhead box motif, and red region defines the zinc finger motif. Most of the KLF proteins contain three zinc finger motifs close at the carboxyl-terminal edge, while KLF9, KLF13, KLF14, and KLF16 contain zinc finger motifs more positionally shifted to the middle part of the protein indicated by the double arrow. (**B**) Multiple sequence alignment of three zinc fingers and preceding amino-terminal amino acid residues of Drosophila melanogaster (Fly) Buttonhead, Krüppel, human SP1/KLF family proteins. The first three zinc finger sequences are shown for the Fly Krüppel protein. Alignment was generated by MAFFT, ClustalW, and visual inspection. Buttonhead box motifs (Btd motif) are highlighted by green color. Cystein and Histidine residues of the zinc finger motifs are highlighted by yellow and blue color, respectively.

Kruppel-like factors were initially identified as C2H2 type zinc finger proteins that contain amino acid sequences resembling the Drosophila segmentation gap gene Krüppel (Figure 1) [7]. Kruppel-type zinc fingers contain a consensus amino acid sequence (Y/F)X**C**X2**C**X3FX5LX2**H**X3**H**TGEKP, in which X can be any amino acid (Cys and His residues conserved in the C2H2 class written in bold) [8,9,10]. The consensus features of the Kruppel family of zinc finger genes include an H-C link (amino acid sequence HTGEKP(Y/F)XC connecting the His of one finger to the Cys of the next) [7]. As conservation is observed in the zinc finger amino acid sequences, as well as in the interfinger spacer domain, this sequence has been called the Krüppel motif (Figure 1A,B). The Krüppel motif is also observed in molecules belonging to the Sp1 (Figure 1B) and GLI families [11,12,13,14]. In fly embryos, the *Krüppel* gene is intimately involved in the anterior–posterior axis and body segment formation [15]. Through the positive and negative transcriptional effects of maternally coordinated genes (consisting mainly of bicoid and nanos), gap genes are zygotically transcribed at specific spatial domains. In particular, the expression domain of *Krüppel* is determined by the maternally derived bicoid, which is expressed anteriorly, and nanos, which is expressed posteriorly. The interactions between these molecules coordinate to form a robustly local Krüppel expression domain. Significantly, remarkable alterations in gap gene expression, together with the secondary pair-rule genes, were observed in Krüppel mutant flies, indicating a central role played by Krüppel in normal embryonic fly morphogenesis [16].

## 3. Kruppel-like Factors in Vertebrates

The KLF family of proteins are transcription factors that play diverse biological roles in cell differentiation, cell cycle regulation, cellular metabolism, embryonic development, and apoptosis by regulating specific target genes [13,14,17,18,19,20]. KLF belongs to a large group of specificity protein 1 (Sp1)/KLF zinc finger-containing transcription factors (Figure 1A) [14]. These family molecules contain 81 amino acid triple-C2H2 zinc finger DNA-binding domains (DBD) separated by two highly conserved linker sequences that share high similarities to the Drosophila Krüppel protein (Figure 1B) [9,21,22]. As the zinc finger motifs existing in the Sp1 class function as a DBD, biochemical binding studies have shown that they have similar affinities for different guanine-cytosine-rich sequences [23,24]. Concurrently, depending on the specific amino acid residues positioned at the zinc finger DBD, these molecules also show a preference for recognizing the unique alignment of guanine-rich sequences [25,26,27]. After the identification of KLF1 (erythroid KLF; EKLF), which was the first zinc finger molecule to contain high similarities to Sp1, many KLFs have been identified in various vertebrate species [27,28]. Importantly, Sp1 clearly differed from KLFs in the presence of the buttonhead (Btd) box domain just 5′ of the KLF-DBD (Figure 1) [29,30,31]. The Btd box domain is a unique alignment present in the Drosophila buttonhead protein (Figure 1B) [30,32]. There are currently 17 KLF family molecules in humans and mice based on the following criteria: (1) the molecule contains a KLF-type DBD without the Btd motif and (2) the presence of only three zinc fingers (Figure 1). As described above, the KLF family plays diverse physiological roles, including skeletal development and maintenance, which are the main focus of this review. Accumulating evidence has also demonstrated that KLFs play crucial roles in the etiology and progression of many mammalian diseases [20]. These include hematopoietic disorders, cardiovascular diseases, gastrointestinal disorders, and bone-related disorders.

## 4. Roles of KLFs in Normal Bone Physiology (Table 1)

### 4.1. KLF1

KLF1 was initially called erythroid KLF (EKLF) because of its crucial role in red cell development and fetal-to-adult hemoglobin switching [33]. KLF1 has been shown to be specifically expressed in erythroblasts in the fetal liver and adult bone marrow [34,35]. Heterozygous KLF1 has been identified in patients with congenital dyserythropoietic anemia type IV [36,37]. These patients display growth defects; however, this is considered a secondary effect of malnutrition and not cell-autonomous effects of KLF1 in bone cells. It has also been reported that some patients with congenital dyserythropoietic anemia type I show congenital skeletal defects, such as missing digits; however, this condition is not caused by KLF1 mutation [38,39]. To date, there is no evidence of the cell-autonomous involvement of KLF1 in bone cell development or maintenance.

### 4.2. KLF2

KLF2 is closely related to KLF1 and is necessary for β-globin gene expression [40]. In contrast to KLF1, which is exclusively expressed in erythroid cells, KLF2 is dynamically expressed in various tissues, including skeletal cells, during embryogenesis [41]. KLF2 knockout animals were embryonic lethal around embryonic day 13 and showed severe abdominal hemorrhage accompanied by abnormal craniofacial morphogenesis [42].

In osteoblasts, KLF2 has been shown to enhance differentiation by upregulating *Runx2,* which is known as one of the master regulators of osteoblast differentiation [43,44]. Osteogenic MC3T3-E1 cell differentiation and mineralization were promoted by KLF2 by increasing Runx2 mRNA and protein levels. In contrast, KLF2 has been shown to negatively regulate RANKL-induced osteoclast differentiation of monocytes in vitro [45]. As conventional *Klf2* knockout animals are embryonically lethal during the mid-developmental stage, the conclusive positive in vivo function of Klf2 in skeletal maintenance needs further investigation.

### 4.3. KLF3

**Figure 2:**
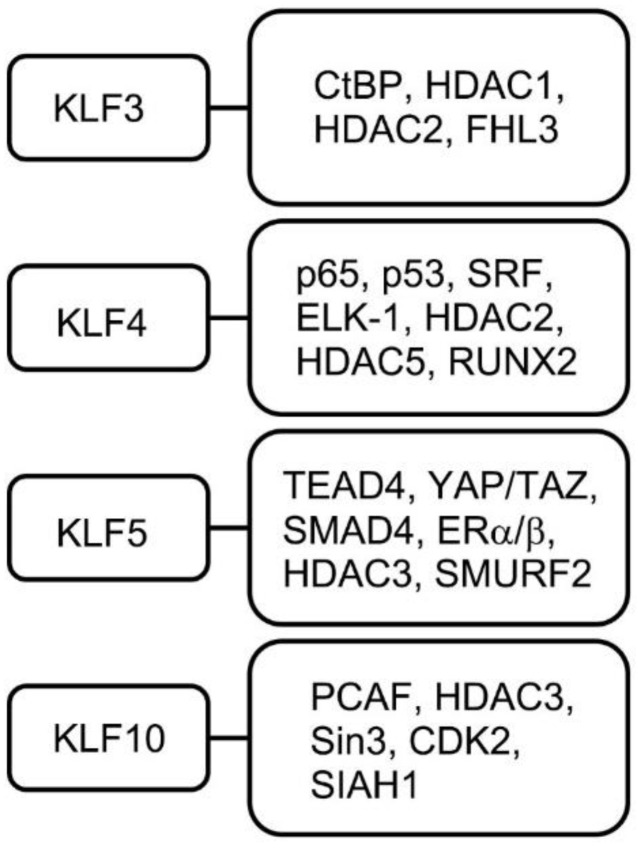
Physical and functional association partner molecules of KLF proteins intimately involved in differentiation of skeletal cells. KLF3 [49,50,51], KLF4 [55,56,61,62,63,64], KLF5 [73,74,75,76,77], and KLF10 [110,111] interacting proteins are shown in the right box of each molecule.

KLF3 inhibition by mutated lncRNA Reg1cp results in human high bone mass syndrome [46]. Reg1cp binds directly to KLF3 and inhibits its activity in vitro. In vivo, mutant Reg1cp increased the formation of CD31^hi^Emcn^hi^ endothelium in the bone marrow by repressing KLF3 transcriptional activity. This was strongly evidenced by the increased number of CD31^hi^Emcn^hi^ vessels and increased bone formation in endothelial cell-specific *Klf3* knockout mice [46]. These endothelial cells appear to couple angiogenesis and osteogenesis, leading to increased bone mass. Bone marrow stromal cell-derived exosomal miR-21-5p can enhance the proliferation and differentiation of osteoblasts [47]. miR-21-5p in exosomes can directly bind to the 3′-UTR of *KLF3* and downregulate its expression, leading to increased osteoblast activity. Another miRNA, miR-20a-5p, has been shown to promote adipogenic differentiation of murine bone marrow stromal cells by targeting KLF3 [48]. Although the development of promising therapeutic strategies targeting miRNAs is still necessary, the regulation of KLF3 activity in bone marrow stromal cells might be one of the ways to increase bone mass while repressing adipogenesis. A number of cofactors have been identified in skeletal and nonskeletal cells for KLF3 functioning as a transcriptional repressor (Figure 2) [49,50,51].

### 4.4. KLF4

KLF4 is expressed in a tissue-restricted manner and is expressed in osteoblasts [52]. *Klf4* conventional knockout mice are perinatally lethal [53]. Kim et al. used an osteoblast-specific Col1a1-Cre deleter strain [54] to generate conditional *Klf4* knockout mice to examine bone phenotypes [55]. They found that osteoblast-specific KLF4 knockout mice showed markedly increased bone mass, despite increased osteoclast differentiation. Overexpression of KLF4 in osteoblasts attenuated osteoblast differentiation and mineralization in vitro [55,56]; at the same time, KLF4 in osteoblasts attenuated osteoclast differentiation in a coculture system of bone marrow cells and osteoblasts [55,57]. Repressed osteoclast maturation was also observed in transgenic mice expressing KLF4, specifically in osteoblasts [57]. KLF4 is physically and functionally associated with Runx2 (Figure 2), which implies that KLF4 functions in a bone-specific manner, although its wide expression has been observed in numerous tissues. KLF4 seems to regulate osteoblast function in numerous ways, including (1) physical and functional association with the master regulator of osteogenesis Runx2 [55,56], (2) blocking vitamin D receptor (VDR) transcriptional activity [55], (3) affecting ciliary-initiated Hedgehog signaling [58], and (4) modulating the expression of the xenobiotic nuclear receptor [59]. Although KLF4 is expressed in osteoclasts at a moderate level, KLF4 does not show cell-autonomous effects on osteoclast differentiation [55]. Significantly, when SP7-cre was used to target osteoprogenitors to delete KLF4 under conditional heterozygotic conditions, these mice showed fewer osteoblasts, reduced bone formation, and significantly smaller body size [60]. These observations indicate that KLF4 functions in skeletal cells in a stage-dependent manner to regulate the differentiation status of osteoblasts and indirectly regulates osteoclast differentiation in a non-cell-autonomous manner to fine-tune the developmental status of the entire skeleton. KLF4 physically and functionally associates with various molecules to play these context- and tissue-dependent roles for normal skeletal development (Figure 2) [61,62,63,64,65].

KLF4 regulates dentinogenesis, which is the formation of another hard tissue (dentin) in the body. KLF4 promotes odontoblastic differentiation of dental papilla mesenchymal cells through the regulation of dentin matrix protein 1 (DMP1) and modulation of transforming growth factor beta TGF-β signaling pathways [66,67,68].

### 4.5. KLF5

KLF5 is a ubiquitously expressed molecule that is predominantly expressed in the skin, digestive tract, kidneys, and urinary bladder [69]. *Klf5* conventional knockout mice died very early in utero before E8.5, indicating that Klf5 plays an indispensable role in early embryogenesis [70]. In bone cells, KLF5 is expressed in developing chondrocytes and osteoblasts, but is not detected in osteoclasts [71]. Klf5+/− mice showed retarded skeletal growth during the perinatal period, but caught up during the early postnatal period. Cartilage matrix degradation was severely impaired, causing delayed primary ossification center formation in the long bones [71]. The possible role of KLF5 in osteoblasts has not been investigated in detail. There is a study suggesting non-cell-autonomous roles of KLF5 in osteoclastogenesis [72]. Numerous cofactors have been identified in a cell- and tissue-context manner that partly explain the indispensable roles played by KLF5 (Figure 2) [73,74,75,76,77,78].

### 4.6. KLF6

KLF6 is a ubiquitously expressed molecule that regulates growth arrest and functions as a tumor suppressor [79]. Gene knockout of *Klf6* is embryonically lethal around E12 due to reduced hematopoiesis and poor yolk sac vascularization [80]. The physiological role of KLF6 in bone cells has not yet been investigated in detail.

### 4.7. KLF7

It has been reported that KLF7 is lowly expressed in people with high bone mineral density [81]. By comparing the gene expression profiles of normal and aged senescent human osteoblasts, Chen et al. identified miRNA targeting KLF7 as one of the genes significantly upregulated in aged osteoblasts [82]. However, the roles of KLF7 have not yet been determined. In contrast, osteoclast differentiation was enhanced by KLF7. Mechanistically, KLF7 directly represses heme oxygenase-1 (HO-1) expression, which is known as a negative regulator of osteoclast differentiation [81,83,84,85]. 

### 4.8. KLF8

KLF8 is a ubiquitously expressed molecule, but there have been no reports regarding its physiological effects on bone cells, both in vivo and in vitro. However, KLF8 plays a critical role in oncogenic and epithelial–mesenchymal transformation in various human tumors, including osteosarcoma, the most common and aggressive primary bone tumor [86,87,88,89,90]. This point is further discussed in a later section.

### 4.9. KLF9

Similar to KLF8, KLF9 has thus far not been reported to be involved in bone development and physiology. KLF9 has been shown to be intimately involved in bone pathology, including osteoarthritis and osteosarcoma [91,92,93,94,95]. This is further discussed in a later section.

### 4.10. KLF10

KLF10 was initially identified as an early-response gene in human osteoblasts following TGF-β treatment [96]. Based on this observation, KLF10 was initially named TGF beta inducible early-response gene 1 (TIEG1). KLF10 has important roles in bone biology by modulating a number of crucial signaling pathways, including TGF-β, bone morphogenetic protein (BMP), and estrogen-mediated pathways [97,98,99,100,101]. The indispensable roles played by KLF10 during skeletal development have been underscored by the skeletal phenotype observed in *Klf10* knockout mice [102]. *Klf10* knockout mice show growth defects in body length, delayed long bone growth, and delayed primary ossification center formation [102,103]. Chondrocyte maturation in the epiphyseal growth plate is also suppressed in long bones. Indian hedgehog (Ihh) expression in *Klf10* knockout mice growth plates was severely reduced, which potentially altered the PTHrP-Ihh negative feedback loop [102]. *Klf10* knockout mice show female-specific defects in bone strength and microarchitecture [103,104]. The osteopenic bone phenotype of *Klf10* knockout mice was rescued by sclerostin antibody treatment [105]. In humans, single-nucleotide polymorphisms (SNPs) in *KLF10* are associated with decreased volumetric bone mineral density (BMD) at the femoral neck in men [106]. In vitro studies have shown that KLF10 controls the expression and function of Runx2 and Sp7 (osterix) in osteoblasts [107,108]. In contrast, KLF10 also regulates osteoclast differentiation and survival by mediating NFATc1, Akt serine/threonine kinase (AKT), and extracellular signal-regulated kinases (ERK) pathways, and loss of KLF10 reduces NFATc1 activation, which slows the rate of osteoclast differentiation [109]. In summary, KLF10 displays pleiotropic cell- and non-cell-autonomous roles in skeletal cell differentiation, and numerous cofactors have been identified (Figure 2) [110,111].

### 4.11. KLF11

Similar to KLF10, KLF11 was initially termed as TIEG2 because of its induction after TGF-β stimulation [112]. KLF11 promotes the effects of TGF-β on cell growth control in vitro by influencing the Smad-mediated pathway, which leads to the regulation of apoptosis and cell cycle arrest [113]. *Klf11* knockout mice have been generated, but these mice showed no growth, developmental, or hematopoietic defects [114]. A few reports have shown the possible involvement of KLF11 in bone pathologies; these are discussed in a later section.

**Table 1:** In vivo skeletal phenotypes caused by loss (LOF) or gain (GOF) of function of KLFs.

Factors	Phenotypes	Studies
KLF1	Growth defects (LOF in human; unlikely cell-autonomous actions)	[36,37,38,39]
KLF2	Abnormal craniofacial morphogenesis (LOF in mouse)	[42]
KLF3	High bone mass (LOF in human; by mutated lncRNA)	[46]
KLF4	High bone mass with increased osteoclast differentiation (osteoblast-specific LOF in mouse) Repressed osteoclast maturation (osteoblast-specific GOF in mouse)	[55] [57]
KLF5	Retarded skeletal growth with delayed primary ossification center formation (LOF in mouse)	[71]
KLF6	Remain to be investigated	–
KLF7	High bone mineral density associated with its low expression (in human)	[81]
KLF8	Remain to be investigated	–
KLF9	Remain to be investigated	–
KLF10	Growth defect with suppressed cartilage maturation and primary ossification center formation	[102,103]
KLF11	None	[114]
KLF12	Remain to be investigated (in vitro studies exist)	–
KLF13	Remain to be investigated (in vitro studies exist)	–
KLF14	Remain to be investigated (in vitro studies exist)	–
KLF15	Remain to be investigated (in vitro studies exist)	–
KLF16	Remain to be investigated (in vitro studies exist)	–
KLF17	Remain to be investigated	–

### 4.12. KLF12

Only a few reports have shown that KLF12 affects skeletal cell differentiation. Human umbilical cord blood-derived mesenchymal stem cells (hUC-MSCs) can potentially become osteoblasts in vitro. Zhao et al. showed that the long intergenic non-protein-coding gene *LINC02381* weakens the osteogenic differentiation of hUC-MSCs through the induction of KLF12 [115]. Significantly, KLF12 was able to repress transcription of *Wnt4*, which has been shown to prevent skeletal aging and pathological inflammation [116].

### 4.13. KLF13

KLF13 is ubiquitously expressed in many tissues, but a particularly strong expression is observed in the lymph nodes, thymus, and heart [117]. Lavallee et al. showed that KLF13 is indispensable for cardiac development and is functionally associated with cardiac GATA factors, which are cardinal molecules for normal cardiogenesis [118]. In bone cells, gene expression profiling after dexamethasone stimulation of osteoblastic MC3T3E1 cell line showed that KLF13 expression was significantly induced, but its functional significance has not been elucidated [119].

### 4.14. KLF14

KLF14 is a maternally expressed imprinted transcription factor [120], and is known to be located at the gene locus related to multiple metabolic disease phenotypes [121]. In human bone marrow stromal cells (hBMSCs), KLF14 is downregulated after osteogenic induction [122]. KLF14 overexpression in hBMSCs induced cell cycle arrest and inhibited osteogenic differentiation. In contrast, inhibiting KLF14 expression activated Wnt3a expression, which activates osteogenic gene expression and differentiation [122].

### 4.15. KLF15

Decreased bone formation is responsible for the pathogenesis of glucocorticoid-induced osteoporosis [123]. Recent evidence has shown that glucocorticoid administration enhances KLF15 expression, which leads to a reduction in osteoblast function [124]. In addition to the negative effect of KLF15 on bone cells, KLF15 enhances adipogenesis by regulating adipogenic gene expression, including peroxisome proliferator-activated receptor gamma (PPARγ) and CCAAT-enhancer-binding protein (C/EBPα) [125,126]. KLF15 has also been shown to enhance chondrogenesis of human MSCs in vitro by enhancing Sox9 expression [127].

### 4.16. KLF16

KLF16 is a ubiquitously expressed molecule, including in the bone marrow, but is known to be highly expressed in the brain tissue [128]. Only a limited number of reports regarding the effect of KLF16 on bone cells have been published. Jang et al. recently reported that KLF16 is downregulated during adipogenic differentiation and negatively regulates adipogenesis by targeting PPARγ [129].

### 4.17. KLF17

KLF17 is exclusively expressed in male testicular cells [130], and no studies on cell-autonomous effects on bone cells have been reported to date.

## 5. Role of KLFs in Osteoarthritis, Osteoporosis, and Osteosarcoma

### 5.1. Osteoarthritis

Osteoarthritis (OA) is a multifactorial, inflammatory, age-related disease characterized by cartilage destruction. OA is the most common form of joint disease, resulting in chronic pain and disability [131]. Progressive destruction of articular cartilage is observed due to disequilibrium between anabolism and catabolism of the cartilage extracellular matrix (ECM) [132]. Evidence showing the involvement of KLFs in OA pathologies is rapidly increasing (Table 2). In 2017, Yuan et al. showed that KLF2 is downregulated in human OA chondrocytes [133]. In an in vitro study using IL-1β to mimic OA cartilage, they showed that KLF2 represses matrix metalloproteinase 13 (MMP13) expression induced by IL-1β. Soon after, several KLFs were shown to be downregulated in human OA cartilage through genome-wide or candidate approaches [134,135,136,137,138]. These include KLF2, KLF4, KLF5, KLF9, KLF10, KLF11, and KLF15. KLF2 suppresses cartilage matrix destruction by decreasing the production of reactive oxygen species (ROS) [139]. KLF4 mediates the protective effects of simvastatin on articular chondrocytes [140]. KLF4 strongly induces cartilage collagen genes and proteoglycan-4 (PRG4) [140,141] and represses the expression of catabolic genes [141]. The efficacy of KLF2 and KLF4 induction was also demonstrated by the reduced severity of OA-associated symptoms in a mouse OA model [141]. Activation of the TGF-β pathway or inactivation of the Janus kinase 2 (JAK2)/signal transducer and activator of the transcription 3 (STAT3) pathway by KLF4 have also been suggested as mechanisms for the amelioration of OA symptoms [138,142].

KLF5 has been shown to be downregulated in human OA samples [136,141]; however, the exact role played by KLF5 in OA pathogenesis is still controversial. Shinoda et al. showed that KLF5 causes cartilage degradation through the strong induction of MMP9 [71]. The authors used the surgical OA model in Klf5+/− mice, which, however, did not show any difference when compared to the control mice [71]. Early growth response 1 (EGR1), which is highly expressed in OA cartilage and known to accelerate cartilage degradation, activates KLF5 activity by preventing KLF5 degradation [136]. Contrarily, KLF5 has been shown to protect against chondrocyte damage caused by IL-1b in certain contexts [143]. Kawata et al. also reported that KLF5 was downregulated in human OA samples [141].

The role of KLF9 in OA is still not well understood, but Zheng et al. reported that KLF9 may be related to the suppression of immune cell infiltration [95].

KLF10 and KLF11 are downregulated in human OA samples [134]. However, there are conflicting reports showing that KLF10 and KLF11 are increased in human OA samples. Zheng et al. showed that KLF10 was upregulated in cartilaginous tissue of OA patients as well as in the cartilage of an OA mouse model [144]. KLF10 overexpression in human chondrocytes inhibits proliferation and migration, suggesting possible negative effects on articular chondrocyte maintenance. Han et al. showed that KLF11 is highly expressed in the cartilage tissue of OA patients and in IL-1b-induced human chondrocytes [145].

KLF15 was significantly downregulated in chondrocytes from OA patients [134,135]. An in vitro study has shown that TNF-α acutely represses KLF15 expression in chondrocytes, and KLF15 attenuates the expression of MMP3 induced by TNF-α stimulation [135].

### 5.2. Osteoporosis

Osteoporosis is a systemic chronic pathology that requires long-term management after disease onset. Most osteoporotic patients are elderly; however, similar pathological conditions can be observed in patients undergoing long-term therapeutic glucocorticoid treatment (glucocorticoid-induced osteoporosis—GIO) [123]. The skeletons of patients show low bone mass and microstructural bone quality [146]. This deterioration causes osteoporosis patients to suffer from a high incidence of fragility fractures [147,148]. In severe cases, treatment with bisphosphonates, denosumab, teriparatide, or abaloparatide is essential to prevent femoral or vertebral fractures [146,149]. However, because of several limitations, as well as the high cost of these medicines [146], it is important to identify novel druggable molecular or cellular targets to tackle this difficult disease. Recent evidence has shown that several KLF family molecules are intimately involved in the onset or progression of osteoporosis, which is supported by both human and animal studies (Table 2).

The KLF1 E325R mutation is associated with congenital dyserythropoietic anemia, which causes severe hemolytic anemia in patients [150,151]. Patients with hemolytic anemia are known to have a significantly high risk of developing early onset osteopenia [152], and an increased risk of nonvertebral osteoporotic fractures in elderly men [153]. As the cell-autonomous roles played by KLF1 in skeletal cells are still unknown, these observations could be due to the nonskeletal cell-autonomous roles played by KLF1.

KLF3 inhibition by the mutated lncRNA Reg1cp has been shown to cause human high bone mass syndrome [46]. The mutant Reg1cp directly binds to KLF3 and inhibits its activity. Animal studies have shown that vascular endothelial-specific *Klf3* knockout (KO) mice have increased bone mass, similar to that observed in humans [46]. An intimate relationship between angiogenesis and osteogenesis has been shown earlier [154,155,156] and dysregulation of vasculature is associated with many bone diseases, including osteoporosis [156]. A natural compound, ophiopogonin D, has been identified as a KLF3 inhibitor, and administration of this compound to wild-type animals increased bone mass, raising the intriguing possibility of KLF3 as a therapeutic target [46].

In vivo observations showed that KLF4 plays a crucial role in bone homeostasis in a stage-dependent manner. When KLF4 was conditionally deleted in osteoprogenitor cells using the Sp7-Cre deletion strain [157], mice displayed reduced osteoblastogenesis, resulting in low bone mass and reduced growth even under heterozygotic conditions [60]. However, when Klf4 was conditionally deleted in osteoblasts using the Col1a1-Cre strain [54], this strain showed the opposite phenotype, resulting in markedly high bone mass [55]. Klf4 is known as one of the factors for cellular reprogramming [158], but is considered a strong cell cycle repressor [159]. Klf4 has multifaceted functions, depending on numerous contexts. To further understand the stage-dependent functions of Klf4 in osteoblast-lineage cells, it is important to investigate skeletal phenotypes using other Cre deleter strains such as Ocn-Cre [160], Dmp1-Cre [161], Prrx1-Cre [162], Dermo1-Cre [163,164], and Lepr-Cre strains [165].

The *KLF10* gene was found to be significantly reduced in osteoporotic patients [166]. Another quantitative trait locus (QTL) study showed that the *KLF10* locus is associated with volumetric cortical bone mineral density [106]. In ovariectomized rats, KLF10 expression is reduced in the femoral tissue [167]. Consistent with these observations, *Klf*KO mice show an osteopenic phenotype, resulting in reduced bone strength [103]. However, these skeletal phenotypes are only observed in females [103,104]. The causes of the gender-specific phenotypes observed in *Klf10* knockout mice are still unclear. KLF10 is known to be induced by estrogen receptor-mediated signals; this action may, at least partly, underlie the gender-specific phenotypes [168]. As described above, several genes in osteoblast-lineage cells have been proposed as targets of the effects of estrogens, in which KLF10 is included [101]. Previous reports have shown that the effects of estrogens and androgens on bones are largely caused by their influences on the generation and lifespan of osteoblasts, osteocytes, and osteoclasts. The gender-specific differences of the *Klf10* knockout mice phenotypes could be examined further from these points.

KLF15 is one of the target genes of glucocorticoid receptor signaling and is intimately involved in tissue adipogenesis [169]. Recently, KLF15 was shown to induce GIO by enhancing marrow adipogenesis [126]. Tanshinol, an aqueous polyphenol isolated from Saliva miltorrhiza Bunge [170], exerts protective effects against GIO via unknown mechanisms [171,172]. Recent reports have identified that tanshinol prevents glucocorticoid-induced bone loss by blocking KLF15 activation [124,126].

### 5.3. Bone Tumors

KLF expression and function are altered in a large number of human cancers, and KLFs regulate cancer cell proliferation, apoptosis, metastasis, the tumor microenvironment, and cancer stem cells [173]. Individual KLFs can have either exclusive or redundant functions. Furthermore, several molecular switches that completely change the functions of KLFs in cancer have been identified [174,175,176,177,178]. The context-dependent functions of KLFs remain to be explored. In this section, we summarize the involvement of KLFs in osteosarcoma, which is a major primary bone malignant tumor with a poor prognosis (Table 2).

Osteosarcoma (OS) is the most common malignant bone tumor, and is thought to be derived from bone-forming mesenchymal stem cells. OS accounts for almost 20% of all primary malignant bone tumors worldwide [179,180]. Several KLFs have been reported to be intimately involved in OS proliferation, migration, invasion, and metastasis, as well as OS cancer stem cell regulation.

KLF2 expression is frequently diminished in various malignancies and is considered a tumor suppressor. KLF2 plays a role in growth inhibition and has proapoptotic and antiangiogenic roles. Silencing of KLF2 occurs in OS cells through transcriptional repression mediated by the polycomb group protein enhancer of zeste homolog 2 (EZH2) [181]. The recently discovered circular RNA CircLRP6 was also shown to promote OS progression through the inhibition of KLF2 [182].

KLF4 expression is highly associated with human OS cancer stemness [183]. Overexpression of KLF4 in OS cells increased sphere-forming potential, enhanced stemness gene expression, enhanced chemotherapy resistance, and increased metastatic potential [183]. Adriamycin treatment is known to cause chemotherapy resistance, owing to the enhanced stemness phenotype in OS cells [184]. OS cells expressing high levels of KLF4 accumulated after adriamycin treatment and were considered a key factor for adriamycin-induced stemness. Simvastatin administration significantly reduced KLF4 expression and prevented the adriamycin-induced enhancement of metastasis and cancer stemness in OS cells [184].

KLF8 and KLF11 have also been shown to be involved in OS cancer stem cell induction [185,186]. KLF8 is highly expressed in OS tissues and highly enriched in CD133-positive cancer stem cell populations [185]. This was shown to be caused by KLF8 activating Sox2 transcription via downregulation of miR-429 [185]. The oncogenic roles of KLF8 have been shown in other studies that showed the involvement of KLF8 in epithelial–mesenchymal transition (EMT) processes [86,88,89,187] and chemotherapy resistance [188,189]. KLF11 has previously been shown to play a crucial negative role in cancer stem cells in non-OS tumors [190]. Through genome-wide screening, Wang et al. recently identified KLF11 as a top-hit suppressor molecule in OS cancer stem cell induction [186]. Mechanistically, KLF11 was associated with yes-associated protein (YAP)/TEA domain family member 1 (TEAD) at adjacent DNA sites, where KLF11 suppressed YAP/TEAD functions by recruiting the SIN3 transcription regulator family member A (SIN3A)/histone deacetylase (HDAC) repressor complex [186]. KLF11 was epigenetically silenced in OS cancer stem cells (CSCs), causing sustained YAP/TEAD activation. KLF11 can be activated in OS cells by thiazolidinedione, a drug that is already used for the treatment of type II diabetes [191].

KLF5 is highly expressed in human OS cells [192]. KLF5 appears to promote OS cell proliferation, invasion, and metastasis via inducing miR-487a [192]. Significantly, a small molecule inhibitor of KLF5, ML264, could induce cell cycle arrest, migration, and invasive capabilities of OS cells [193].

KLF6 is considered a tumor suppressor in various tumors and acts by upregulating p21 and downregulating B-cell lymphoma 2 (BCL-2) [194]. KLF6 is downregulated in OS cells, and KLF6 represses OS cell proliferation and invasion in vitro [194].

KLF9 is downregulated in human OS tissues and is known to block progression of OS cells [94]. Repression of KLF9 in OS cells are induced by several microRNAs and CircRNAs [91,93].

**Table 2:** Studies of KLFs in bone pathologies.

	Factors	Conclusion	Studies
Osteoarthritis	KLF2 KLF4 KLF5 KLF15	Downregulated in OA patients Represses IL1β-induced MMP13 expression Suppresses ROS production Downregulated in human OA samples Induces PRG4 and collagen genes Downregulated in human OA samples Downregulated in human OA samples	[133,134,135,136,137,138,139] [140,141] [136,141] [135,136]
Osteoporosis	KLF3 KLF4 KLF10	Endothelial cell-specific KO mice show high bone mass Osteoblast-specific KO mice show high bone mass Osteoprogenitor-specific KO mice show low bone mass Downregulated in osteoporotic patients Downregulated in ovariectomy rats Female Klf10 KO mice show reduced bone strength	[46] [54,60] [103,166,167]
Osteosarcoma	KLF2 KLF4 KLF5 KLF6 KLF8 KLF9 KLF11	Downregulated in OS cells in vitro Induces cancer stemness Highly expressed in OS cells ML264 (KLF5 inhibitor) induces OS cell activation Downregulated in OS cells Highly expressed in OS samples Induces cancer stemness Downregulated in OS cells Downregulated in OS samples Suppresses cancer stem cell induction	[181] [183,184] [192,193] [194] [185,186] [194] [186,191]

## 6. Future Perspectives

The understanding of the role of KLF family molecules has deepened during this decade. However, many aspects need to be investigated further to understand how each member of the KLF protein family regulates gene expression in a cell- and tissue-specific manner. In particular, less-studied KLF family molecules are yet to be fully explored. This goal could be partly achieved by expanding the investigation of cofactors of each KLF protein in various cell types (Figure 2). It is important to reveal the transcriptional complex in which each KLF participates to obtain the overall picture to classify it as a KLF protein complex. This information will enable researchers to predict whether family proteins possess synergistic or antagonistic properties. To introduce KLF as a therapeutic target for specific bone pathologies, we need to understand the functional redundancies of each KLF in disease onset or progression. Finally, as some KLF knockout animals are embryonic or perinatally lethal, the possible roles played by KLFs in human diseases need to be carefully analyzed.

## Data Availability

Not applicable.
